# Slackline Training in Children with Spastic Cerebral Palsy: A Randomized Clinical Trial

**DOI:** 10.3390/ijerph17228649

**Published:** 2020-11-21

**Authors:** Lucía González, Juan Argüelles, Vicente González, Kristian Winge, Marta Iscar, Hugo Olmedillas, Miguel Blanco, Pedro L. Valenzuela, Alejandro Lucia, Peter A. Federolf, Luis Santos

**Affiliations:** 1 Spanish Confederation of People with Physical and Organic Disability (COCEMFE), 33204 Asturias, Spain; vdiez65@hotmail.com; 2 Department of Functional Biology, University of Oviedo, 33006 Oviedo, Spain; jal@uniovi.es (J.A.); olmedillashugo@uniovi.es (H.O.); 3 Medical Service of the Community of Cabo Peñas, 33440 Asturias, Spain; vdiez61@gmail.com; 4 Novo Nordisk Foundation, 2900 Hellerup, Denmark; KWI@novo.dk; 5 University Central Hospital of Asturias (HUCA), 33011 Asturias, Spain; martaiscar@gmail.com; 6 Catholic University of Valencia, 46001 Valencia, Spain; blanco.traba.miguel@gmail.com; 7 Department of Systems Biology, University of Alcalá, 28805 Alcalá de Henares, Spain; pedro.valenzuela92@gmail.com; 8 European University of Madrid (Faculty of Sport Sciences) and Research Institute Hospital 12 de Octubre (‘i+12’), 28041 Madrid, Spain; alejandro.lucia@universidadeuropea.es; 9 Department of Sport Science, University of Innsbruck, 6020 Innsbruck, Austria; peter.federolf@uibk.ac.at; 10 Department of Physical Education and Sport, University of León, 24007 León, Spain; 11 Performance and Health Group, Department of Physical Education and Sport, University of A Coruña, 15179 A Coruña, Spain

**Keywords:** neuro-disability, motor disorders, rehabilitation, exercise, non-conventional balance therapy

## Abstract

Objective: To assess whether a slackline intervention program improves postural control in children/adolescents with spastic cerebral palsy (CP). Design: Randomized controlled trial. Setting: Patients’ association. Participants: Twenty-seven children/adolescents with spastic CP (9–16 years) were randomly assigned to a slackline intervention (*n* = 14, 13 ± 3 years) or control group (*n* = 13, 12 ± 2 years). Intervention: Three slackline sessions per week (30 min/session) for 6 weeks. Main outcome measures: The primary outcome was static posturography (center of pressure—CoP—parameters). The secondary outcomes were surface myoelectrical activity of the lower-limb muscles during the posturography test and jump performance (countermovement jump test and Abalakov test). Overall (RPE, >6–20 scale) rating of perceived exertion was recorded at the end of each intervention session. Results: The intervention was perceived as “very light” (RPE = 7.6 ± 0.6). The intervention yielded significant benefits on static posturography (a significant group by time interaction on Xspeed, *p* = 0.006) and jump performance (a significant group by time interaction on Abalakov test, *p* = 0.015). Conclusions: Slackline training improved static postural control and motor skills and was perceived as non-fatiguing in children/adolescents with spastic CP.

## 1. Introduction

Cerebral palsy (CP) is a permanent disorder affecting the development of movement and posture that causes activity limitations and is attributed to non-progressive disturbances to the developing fetal or infant brain [1]. Fatigue is another reality in the lives of children with CP [2] including those who experience spasticity (especially in lower leg muscles during walking) [3], which is the most common clinical expression of CP [4]. Rehabilitation therapies are therefore needed to counteract the aforementioned functional limitations.

Slacklining consists of walking or maintaining balance on a polyester band placed between two anchor points. The activity itself is a specialized open motor skill, with demands on muscle coordination and postural stabilization with respect to variable external conditions and their anticipation [5]. It allows high movement variability, provides a small, non-fixed base of support, elicits fast medio-lateral perturbations to the body and induces high challenges for the postural control [6]. Previous studies on slacklining have shown that this training modality improves postural stability in young healthy adults and athletes [7,8,9]. Other studies reported that balance tasks performed on a slackline require minimal energy expenditure [10] and result in low levels of perceived fatigue in athletes or patients with Parkinson’s disease [7,8,11]. Given these benefits, slackline training might improve balance and consequently motor performance in patients with spastic CP. To the best of our knowledge, however, no study has analyzed the effects of a slackline intervention program in this patient population.

The main aim of this study was to analyze the effects of a slackline intervention program on the postural and motor skills of children with spastic CP. It was hypothesized that slacklining would yield significant benefits including an improvement in patients’ postural control.

## 2. Materials & Methods 

### 2.1. Trial Design

The present randomized controlled trial (clinicaltrials.gov ID: NCT03486483, date of registration 03/04/2018; https://clinicaltrials.gov/ct2/show/NCT03486483) complies with the recommendations of the consolidated standards of reporting trials (CONSORT) statement. The study was conducted in accordance with the updated declaration of Helsinki and was approved by the Regional Clinical Research Ethics Committee of the Principality of Asturias, Spain (No. 53/2014). Patients provided assent and their parents’ written informed consent to the research and to publication of the results.

### 2.2. Participants

Participants were included if they met each of the following criteria: (1) diagnosed with spastic CP and level I or II on the Gross Motor Functional Classification [12]; (2) aged 9 to 16 years; (3) do not present significant intellectual disability (able to understand study’s procedures); (4) able to stand for ≥2 min and to walk ≥10 m without assistance; and (5) not having used a slackline before. Individuals were excluded if: (1) they had undergone neuro or ortho surgery within the year prior to the start of the study, botulinum toxin injection or intrathecal pump implantation within six months prior to the start of the study, or rotational osteotomies; (2) they showed gait deviations limited by musculo-sketal contracture; or (3) they presented with specific medical or orthopedic conditions that precluded doing physical exercise. All participants had received physical therapy from early childhood (mainly aimed at improving postural control, balance, coordination, decreasing the tension of the muscles groups that express hypertonia and preventing the muscles groups shortening, 3–4 days per week for 30–40 min). This routine was kept through the present study duration and was supervised for the patients’ habitual therapist (who was not informed of the study’s goals). Patients and their parents were instructed to strictly follow their usual medications and drug regimens throughout the study.

The study’s sample size was determined according to Cohen’s statistical power analysis for analysis of variance (ANOVA) design [13]. This process was performed using R software (www.r-project.org, version 3.3.1., 2016.06.21) setting a Cohen’s effect size [13] (Cohen’s f) of 0.50, a maximum significance level of 0.05, and a minimum power of 0.80. The resulting sample size was 17 participants in each group.

### 2.3. Intervention

Participants were randomly assigned to an intervention or control group using a randomization algorithm (http://www.graphpad.com/quickcalcs/randomize1.cfm), which was applied twice to ensure full randomization. Participants in both groups followed usual care throughout the study duration, and the intervention group also participated in 18 slackline intervention sessions within 6 weeks (three 30-min sessions per week on non-consecutive days).

Each intervention session included a 10-min warm-up period with stretching of the main locomotive system muscles, followed by 15 min of slackline tasks, and ending with a 5-min cool-down phase including active and static stretching. Slackline tasks consisted of maintaining simple body postures in the bipedal position, taking forward and backward steps and performing 90º turns (see Table 1 for more details). All intervention exercises were supervised for the same therapist, who was not the patients’ habitual one (he/she was not informed of the study’s goals). All exercises were performed with participants barefooted on a slackrack (Gibbon™ Slackrack 300, ID Sports, Stuttgart, Germany; height: 30 cm; length: 3 m; width: 5 cm).

Patients in the intervention arm were required to complete at least 17 of the 18 scheduled intervention sessions (i.e., adherence >90%) for their data to be included for analysis.

### 2.4. Outcome Measures

The primary study outcome was static posturography and the secondary outcomes myoelectrical activity and jump performance. Testing was conducted by the same group of evaluators, who were not informed to the study’s goals, before and after the 6-week intervention. Pre- and post-tests assessments were done within 72–96 h of the first and last intervention session, respectively.

#### 2.4.1. Static Posturography 

The Speed center of pressure (CoP) parameters were assessed using a 30-s bipedal standing support test with open eyes on a firm surface (Speed: postural reactions to maintain balance; Xspeed and Yspeed: postural reactions to maintain balance in the medio-lateral and antero-posterior directions, respectively). A foot-scan baropodometric platform (T-plate, Medicapteurs; Balma, France) was used to acquire data, which were registered and analyzed with specific software (T-plate 3.0, Medicapteurs). A sampling frequency of 100 Hz was used for data collection. All the tests were performed with the participants barefooted, with a 17-cm stance width and their hands held akimbo. Participants were asked to ‘stand as still as possible’, as well as to rest their gaze on a target (10 cm^2^ circle) that was elevated 1.65 m and situated 2.5 m from the platform. Three attempts interspersed with 1-min rests were recorded and data were averaged for statistical analyses [14]. An attempt was considered invalid and repeated if participants grasped the test assistants (two were available to prevent falls). 

#### 2.4.2. Myoelectrical Activity

Patients’ surface electromyographic (SEMG) activity (in µV) of the lower limbs was recorded during the static posturography test (Mega Biomonitor ME6000 8-channel system, Mega Electronics Ltd.; Kuopio, Finland). The signal was recorded with a sampling frequency of 1000 Hz and was processed with band pass filtering of 8–500 Hz. Ag/AgCl sensor electrodes (Medicotest Blue, M-00-S; Copenhagen, Denmark) were used with an inter-electrode distance of 2 cm. SEMG data were stored and analyzed using MegaWin software for Windows (2.4 version) (Mega Electronics Ltd.). The SEMG activity from the three major postural leg muscles (*soleus*, *tibialis anterior* and *peroneus longus*) [15] was analyzed during CoP tests. This process was developed according to the European recommendations for SEMG [16]. All SEMG analyses were performed unilaterally, on the patients’ most affected limb. Impedance, which was accepted below 5 kΩ, was monitored before and after SEMG recordings (Falk Minow Services, SIGGI II-Impedance Meter; Herrsching-Breitbrunn, Germany). SEMG amplitudes were quantified using the root mean square mode of the device and processed as a moving average over 100 ms. The SEMG data from the three posturographic tests were averaged and then analyzed. SEMG activity during the tests was expressed as a percentage of the activity attained during a maximum voluntary isometric contraction (%SEMG_MVC_), which had been performed before each testing, attending to the European recommendations regarding SEMG for Non-Invasive Assessment of Muscles [16].

#### 2.4.3. Jump Performance

After a standardized warm-up consisting of 5 min of cycle-ergometer exercise at 100 watts, patients performed a countermovement jump (CMJ) and an Abalakov test on a contact mat (Digitest, Ergojump Digitime 1000; Jyväskylä, Finland). During the CMJ test participants squatted down until reaching a knee flexion of ~90º, and then immediately jumped vertically as high as possible, taking off and landing with both feet at the same time. Participants were told to hold their hands akimbo during the test, and not to bend their knees during the flight or the landing phase in order to avoid an overestimation of the flight time. The Abalakov test was performed similarly to the CMJ except that the upper limbs had freedom of action [17]. Participants performed three attempts at each test interspersed with 1-min rests and data were averaged for analysis. Patients were allowed to familiarize themselves with the procedures before testing.

#### 2.4.4. Perceived Exertion

The overall rate of perceived exertion (RPE) was assessed at the end of each intervention session using the Borg 6 to 20 points scale [18]. The Borg scale assumes a linear function between perceptual, physiological and physical fatigue and is used as a predictor of self-imposed exhaustion. The scale was explained to the patients prior to the program (patients self-rated several times prior to the intervention). 

### 2.5. Statistical Analysis 

All values are shown as mean and standard deviation unless otherwise noted. Data normality and sphericity were evaluated using the Shapiro-Wilks and Mauchly’s tests, respectively. When these were verified, a two-way ANOVA with repeated measures was performed to assess changes in the dependent variables using time (pre- and post-test) and groups (intervention and control) as factors. Conversely, when they were not confirmed the robust tsplit test (equivalent to the two-way ANOVA with repeated measures) [19] was performed. Significance level was set to an alpha of 0.05. The Tukey HSD post-hoc test and the robust rmmcp test were used to determine within-groups differences for the ANOVA and the robust tsplit test, respectively. Effect sizes (Cohen’s f and Rosenthal’s r for normally and non-normally distributed data, respectively) were also computed and considered small (f > 0.1, r > 0.20), medium (f > 0.25, r > 0.50) or large (f > 0.40, r > 0.80) [13,20]. All data were analysed using R software (www.r-project.org, version 3.3.1, 2016.06.21).

## 3. Results 

**Figure 1:**
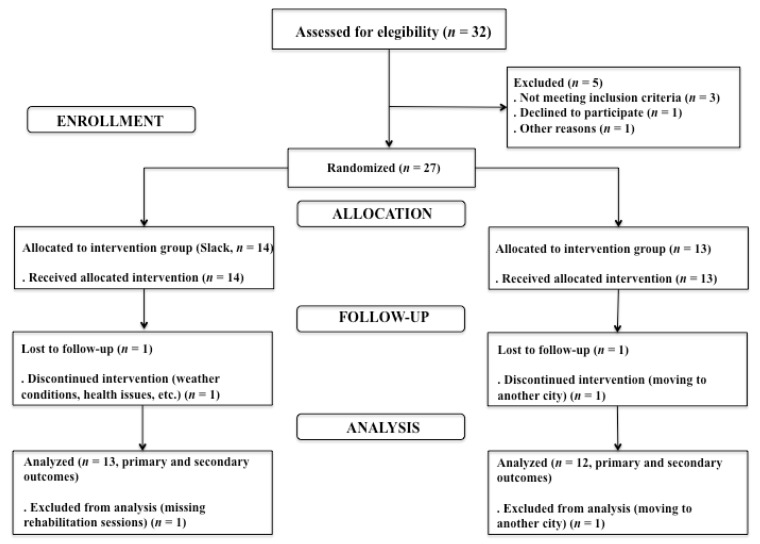
Flowchart of study participants.

The flow diagram of study participants is shown in Figure 1. Twenty-seven patients with spastic CP participated in the study: 14 and 13 assigned to the intervention and control group, respectively. No significant between-group differences were found at baseline (Table 2) and no adverse events were reported during the intervention. 

The intervention group reported a mean RPE value of 7.6 ± 0.6 during the slackline program (corresponding to a perception of “very, very light”). Two significant group by time interactions were found for Xspeed and Abalakov test (*p* = 0.006 and *p* = 0.015, respectively, Table 3) with the slackline intervention, and not the control, inducing significant benefits from baseline. Additionally, another non-significant group by time interaction trend was detected for Yspeed (*p* = 0.077, Table 3) for the intervention group, and not for the control. The slackline intervention, and not the control, also yielded significant between-group benefits for Speed (*p* = 0.041, r = 0.64, Table 3, *p* = 0.057 in the post-test) and Xspeed (*p* < 0.000, r = 0.71, Table 3, *p* = 0.006 in the post-test) and non-significant between-group benefits for Yspeed (*p* = 0.062, Table 3). At the same time, the slackline intervention, and not the control one, yielded significant within-group benefits for Xspeed (*p* = 0.055, r = 0.71, Table 3, *p* = 0.015 in the intervention group) and Abalakov test (*p* = 0.055, f = 1.99, Table 3, *p* = 0.034 in the intervention group). No between-group effects, within-group effects or group by time interactions were observed for any other outcome.

## 4. Discussion

The present study analyzed the effects of a 6-week slackline intervention program on the postural and motor skills of children with spastic CP. Results show that patients who participated in the slackline program increased their static postural control and motor skills, as reflected by an increased jump in performance. Overall, these findings highlight the potential of slackline training as a simple and inexpensive intervention tool in this patient population. In addition, the program was overall perceived as “very light”, which reinforces its applicability. 

The overall improvement in the Speed CoP parameters (with moderate effect sizes) supports the benefits of the slackline intervention on patients’ enhanced postural control, since their reduction represents increases in the ability to maintain an upright stance [21]. Thus, the present findings suggest that the slackline therapy might be effective for enhancing static postural control, which is in line with previous studies performed in non-clinical populations [5,8,22]. To the best of the authors knowledge, the present study is the first to use this type of intervention in patients with CP, but other training/rehabilitation methods have previously proven effective for the improvement of postural control in patients with CP [23,24,25,26,27,28]. According to Paillard [29], adaptations in neurophysiological components (neural circuits and sensory processing), cognitive function relative to body representation in space (cortical regulation), and motor function (muscular command) can explain changes in postural control. Thus, it is hypothesized that these variables (e.g., amelioration of the functional deficits of afferent, particularly somatic fibers) might be involved in the benefits observed in the present study [30].

No changes were, however, observed in the SEMG activity of the three major postural leg muscles during the posturography test. This finding is in agreement with other studies that have analyzed the effects of slackline training effects on the SEMG of several trunk and lower limb muscles in different populations [5,8,22,31,32]. Thus, the existing evidence overall suggests that slackline tasks might not provide a sufficiently high stimulus to elicit changes in SEMG. By contrast, other intervention programs involving balance training have previously yielded positive results in both balance and SEMG in patients with CP [33,34].

The large improvement observed for jump performance (Abalakov test) is also worth noting, as it suggests that slackline training might potentially improve the motor skills of patients with spastic CP. In this regard, however, the scientific literature is equivocal, with all research having been conducted on healthy individuals [4,8,22,35]. The differences between studies might be due to the different populations included (healthy children, healthy active adults, and athletes), the different interventions applied (total duration ranging from 4 to 6 weeks, 2 to 5 sessions per week, and 5 to 60 min per session) and to the different jump tests used as assessment tools (CMJ, Abalakov, etc.) The mechanisms underlying the benefits on jump performance remain to be elucidated. Nevertheless, given that slackline exercises require a high level of coordination of segment movements, which is essential for jump performance [36], it seems plausible to hypothesize that slackline training might improve patients’ general coordination skills.

In agreement with previous studies conducted on healthy individuals [4,8], the intervention was perceived as “very light”. This underscores that the slackline tasks do not yield a fatigue perception, which is of major clinical relevance because patients with CP commonly express high levels of fatigue, especially those who experience spasticity [37]. Thus, the current study’s results suggest that slackline training might be a simple, inexpensive and effective tool to enhance postural control and motor skills in children with spastic CP without inducing fatigue, which holds great potential for development. It can be applied through many different rehabilitation protocols (e.g., short-term protocols with few tasks such as simple static body postures and forward and backward steps using only one line or several, long-term protocols with many tasks such as simple and complex static body postures, forward and backward steps, 90º–180º turns, capturing and throwing objects in static body postures, using only one line or several, etc.), being combined with other rehabilitation balance tools (e.g., all types of stable and unstable surface, etc.) and with other rehabilitation topics (e.g., strength, endurance, etc.). Moreover, under the supervision of professionals, it could also be used in recreation and school settings following the aforementioned protocols, since Slackline is a challenging activity for children, who like to be “on the line”, giving their best and trying to challenge their bodies against gravity.

The present study has some limitations that warrant attention. Notwithstanding that the sample size was not small, it should include more patients. The postural control analysis was assessed through CoP parameters, but a center of mass or a full body assessment [37] might have provided deeper insights. That being said, the patients presented spasticity and muscular atrophy, and thus they might have not been able to exert maximum effort (and consequently maximum SEMG) during the maximum voluntary isometric contraction tests conducted for the SEMG assessment.

Nonetheless, these preliminary findings provide promising data for further investigation. Future studies should present longitudinal designs including detraining and also compare the effectiveness of slackline training with that of other balance training interventions.

## 5. Conclusions

In summary, the present study shows preliminary evidence that a 6-week slackline intervention program improves static postural control and motor skills in children and adolescents with spastic CP (levels I and II of the Gross Motor Function Classification System). The intervention resulted in a low fatigue perception (i.e., perceived as “very light”). Although preliminary, these findings support the role of slackline training as a simple and inexpensive rehabilitation tool in children with spastic CP, with great potential for development.

## Figures and Tables

**Table 1:** Description of the tasks performed during the slackline program.

Weeks	Tasks Description
1st	Task-1: Stand on the band using the right leg as support (with arms and the left leg free). The therapist supplies “major help”. Task-2: Repeat task 1 but using the left leg as support. Task-3: Tandem stance. Patients set the left leg as the rear one. The therapist supplies “major help”. Task-4: Repeat task 3 but using the right leg as the rear one. This set of 4 tasks should be carried out 4 times.
2nd	Task-1: Stand on the band using the right leg as support (with arms and the left leg free). The therapist supplies “minor help”. Task-2: Repeat task 1 but using the left leg as support. Task-3: Tandem stance. Patients set the left leg as the rear one. The therapist supplies “minor help”. Task-4: Repeat task 3 but using the right leg as the rear one. This set of 4 tasks should be carried out 3 times. Task-5: Patients walk 4 forward steps. The therapist supplies “major help”. Task-6: Patients walk 6 forward steps. The therapist supplies “major help”.
3rd	Task-1: Patients walk 4 forward steps. The therapist supplies “major help”. Task-2: Patients walk between 6 and 8 forward steps. The therapist supplies “major help”. Task-3: Patients walk 4 forward steps. The therapist supplies “minor help”. Task-4: Patients walk between 6 and 8 forward steps. The therapist supplies “minor help”. Task-5: Patients walk 4 forward steps. The therapist does not supply help. Task-6: Patients walk between 6 and 8 forward steps. The therapist does not supply help. This set of 6 tasks should be carried out twice.
4th	Task-1: Patients walk 4 forward steps. The therapist supplies “minor help”. Task-2: Patients walk between 6 and 8 forward steps. The therapist supplies “minor help”. Task-3: Patients walk 4 forward steps. The therapist does not supply help. Task-4: Patients walk between 6 and 8 forward steps. The therapist does not supply help. Task-5: Patients walk 4 backward steps. The therapist supplies “major help”. Task-6: Patients walk between 4 and 6 backward steps. The therapist supplies “major help”. This set of 6 tasks should be carried out twice.
5th	Task-1: Patients walk between 4 and 6 forward steps. The therapist does not supply help. Task-2: Patients walk 4 backward steps. The therapist supplies “major help”. Task-3: Patients walk between 4 and 6 backward steps. The therapist supplies “major help”. Task-4: Patients walk 4 backward steps. The therapist supplies “minor help”. Task-5: Patients walk between 4 and 6 backward steps. The therapist supplies “minor help”. Task-6: Patients walk 4 backward steps. The therapist does not supply help. This set of 6 tasks should be carried out twice.
6th	Task-1: Patients walk 4 backward steps. The therapist supplies ‘minor help’. Task-2: Patients walk between 4 and 6 backward steps. The therapist supplies “minor help”. Task-3: Patients walk 4 backward steps. The therapist does not supply any help. Task-4: Patients walk between 4 and 6 backward steps. The therapist supplies “minor help”. Task-5: Patients make 2 90º turns towards right and left side while standing. The therapist supplies “major help”. Task-6: Patients make 2 90º turns towards right and left side while standing. The therapist supplies “minor help”. This set of 6 tasks should be carried out twice.

The workout time of the tasks was always 30 s with 30 s of rest (workout-rest ratio 1:1). “Major help”: therapist places one of their hands over the patients’ lumbar area and the other one over the elbow of the closer arm; “minor help”: refers to a support over one of the patient’s elbows.

**Table 2:** Patients’ baseline characteristics by group.

Characteristic	Intervention (*n* = 14)	Control (*n* = 13)	*p*-Value
Age (years)	13 ± 2	12 ± 2	0.679
Sex (male/female)	7/7	8/5	0.688
GMFCS (I/II)	8/6	8/5	1.000
SCP type (dip/hem)	8/6	7/6	1.000
BMI (kg.m^−2^)	21.2 ± 4.2	19.5 ± 3.1	0.251

Data are shown as mean standard deviation. Abbreviations: BMI, body mass index; GMFCS, Gross Motor Function Classification System; SCP, spastic cerebral palsy; dip, diplegic; hem, hemiplegic.

**Table 3:** Effects of the slackline program on the study’s endpoints.

Endpoints	Group	Baseline	Post-intervention	Group *p*-Value	Time *p*-Value	Interaction *p*-Value	Effect Size
Speed (mm/s) ^b^	Intervention	6.4 ± 3.6	4.1 ± 1.6 *	*p* = 0.041 *	*p* = 0.229	*p* = 0.269	**r = 0.64**
	Control	7.5 ± 2.9	7.8 ± 2.9				
Xspeed (mm/s) ^b^	Intervention	6.2 ± 3.07	2.1 ± 1.9 *	*p* < 0.000 *	*p* = 0.055 *	*p* = 0.006 *	**r = 0.71**
	Control	7.04 ± 3.1	7.6 ± 3.2				
Yspeed (mm/s) ^b^	Intervention	7.2 ± 3.5	4.1 ± 2.8	*p* = 0.062	*p* = 0.345	*p* = 0.077	--
	Control	6.9 ± 4.1	8.2 ± 4.4				
*Soleus* (%SEMG_MVC_) ^b^	Intervention	18.7 ± 9.5	19.4 ± 10.3	*p* = 0.760	*p* = 0.926	*p* = 0.802	---
	Control	20.6 ± 13.4	19.7 ± 11.3				
*Tibialis anterior* (%SEMG_MVC_) ^b^	Intervention	12.8 ± 6.5	13.1 ± 6.5	*p* = 0.372	*p* = 0.218	*p* = 0.692	---
	Control	14.4 ± 7.5	16.6 ± 8.5				
*Peroneus longus* (%SEMG_MVC_) ^b^	Intervention	14.5 ± 8.9	15.1 ± 8.4	*p* = 0.511	*p* = 0.656	*p* = 0.863	---
	Control	16.9 ± 6.4	18.1 ± 6.8				
CMJ (cm) ^a^	Intervention	12.4 ± 4.3	15.2 ± 6.2	*p* = 0.435	*p* = 0.405	*p* = 0.214	---
	Control	11.5 ± 4.3	10.9 ± 3.8				
Abalakov (cm) ^a^	Intervention	15.9 ± 6.2	22.7 ± 7.2 *	*p* = 0.230	*p* = 0.055 *	*p* = 0.015 *	**f = 1.99**
	Control	13.7 ± 5.7	12.7 ± 5				

All values represented as mean standard deviation. * Significant *p*-values (*p* ≤ 0.05) are highlighted in bold. Abbreviations: %SEMG_MVC_, percentage of surface electromyographic activity attained during a maximal voluntary isometric contraction; CMJ, countermovement jump test; f, effect size for parametric statistical assessment; r, effect size for non-parametric statistical assessment. ^a^ Parametric statistical assessment with two-way ANOVA with repeated measures. ^b^ Robust statistical assessment with tsplit test (equivalent to the two-way ANOVA with repeated measures).

## Data Availability

The data that support the findings of the present study are available upon reasonable request.
